# A mixed methods evaluation of an online climate change and health certificate program for working professionals

**DOI:** 10.1186/s12889-025-23477-7

**Published:** 2025-07-03

**Authors:** Daniel Carrión, Saira Prasanth, Ivan Hurtado, Anna Lin-Schweitzer, Maricel Braga, Sammi Munson, Emmanuelle Hines, John Kotcher, Heather Daly-Haney, Edward Maibach, Kathryn Conlon, Lauren Babcock-Dunning, Kristin Timm, Robert Dubrow

**Affiliations:** 1 Department of Environmental Health Sciences, Yale School of Public Health, New Haven, CT USA; 2 Yale Center on Climate Change and Health, Yale School of Public Health, New Haven, CT USA; 3 Department of Chronic Disease Epidemiology, Yale School of Public Health, New Haven, CT USA; 4 FHI 360, New York, NY USA; 5 Department of Social and Behavioral Sciences, Yale School of Public Health, New Haven, CT USA; 6 Harvard T.H. Chan School of Public Health, Boston, MA USA; 7 Department of Environmental Studies and Sciences, Gonzaga University, Spokane, WA USA; 8 RMI, Boulder, CO USA; 9 Center for Climate Change Communication, George Mason University, Fairfax, VA USA; 10 Yale Center for Business and Society, Yale School of the Environment, New Haven, CT USA; 11 Department of Public Health Sciences, UC Davis School of Medicine, Davis, CA USA; 12 Yale School of Public Health, New Haven, CT USA; 13 International Arctic Research Center, University of Alaska at Fairbanks, Fairbanks, AK USA

**Keywords:** Climate change and health, Online education, Public health, Climate adaptation, Climate communications, Continuing education

## Abstract

**Background:**

Climate change is one of the greatest public health challenges of the 21st century, and a diverse cadre of professionals are increasingly pursuing opportunities to learn about this issue. Our goal was to evaluate an online climate change and health (CCH) certificate program for working professionals as a case study.

**Methods:**

We utilized structured and free-text elements from course and overall program evaluations across seven cohorts of participants from 2018 to 2022 for a mixed-methods assessment of the program.

**Results:**

A total of 579 enrollees’ data were analyzed. The program completion rate was 90.0% and participants were diverse in their professional and geographic representation, but disproportionately from North America (82.4%). The program was rated favorably; i.e., 98.6% of participants would recommend the program to others. Qualitative analysis identified themes that were grouped as program strengths and opportunities for improvement. Strengths included (1) valuable curriculum, (2) peer-to-peer learning, and (3) a call to action. Opportunities for improvement included: (1) irrelevant course content, (2) challenges with course structure, and (3) insufficient opportunities for networking.

**Conclusions:**

The evaluations demonstrate the overall importance of educational offerings in CCH. Our case study demonstrated that working professionals in public health, allied health professions, and non-health professions express desire, benefit, and appreciation for educational opportunities on this important intersection. They especially valued strong, relevant course content, professional networking and peer-to-peer learning, and acquisition of effective skills for use in their work. We have used lessons learned from this case study to improve our program and encourage others to use them to improve their programs or to develop new ones. A workforce that is highly educated on CCH issues is essential to confront the existential public health challenge of climate change.

**Supplementary Information:**

The online version contains supplementary material available at 10.1186/s12889-025-23477-7.

## Introduction

It is widely recognized that climate change is one of the greatest public health challenges of the 21st century [1]. Consequently, a growing number of educational offerings are training participants on the nexus of climate change and health (e.g., [2]). Most of these offerings are being integrated into preexisting degree programs, such as incorporating climate change into medical school and nursing curricula and offering a climate change and health track in MPH degrees [3, 4]. However, these offerings are still relatively rare, and most of the current health workforce has received no such training. Many health professionals describe how their limited knowledge is a barrier to further engagement with climate change [5], and have called for more training opportunities [6]. Additionally, making strides on climate change and public health requires professional involvement from multiple sectors, including environment, energy, transportation, buildings, food and agriculture, urban planning, meteorology, economics, law, and others, beyond the health workforce [7]. There also is a critical role for government officials, policymakers, and advocates.

Given the paucity of health and other working professionals with a knowledge-base in climate change and health, in 2018 the Yale Center on Climate Change and Health launched a completely online, 18-week non-degree Climate Change and Health Certificate program to train working professionals located anywhere in the world on the public health challenges of climate change and opportunities for intervention. The program consisted of three consecutive six-week courses: Introduction to Climate Change and Health (Course 1), Climate Adaptation for Human Health (Course 2), and Communicating Climate Change and Health (Course 3).

Course 1 covered a basic introduction to climate science; the impacts of climate change on health, including direct impacts and those mediated by stress placed on human political, economic, and social systems; vulnerability and equity; and an introduction to climate change mitigation and adaptation. The course conducted in depth examinations of heat extremes; storms, floods, and waterborne infections; vector-borne infections; and health-co-benefits of climate change mitigation. Course 2 covered adaptation planning to protect public health; iteration through adaptation planning, implementation, and improvement; vulnerability assessments to identify and prioritize opportunities for adaptation; inter-professional collaborative adaptation efforts; and practical examples of adaptation tools and activities that practitioners can implement in their communities. Course 3 focused on communicating with diverse publics about the health risks of climate change and responses for risk reduction, including individual behaviors and collective actions to advocate for policy changes. The course introduced best practices and examined the specific challenges to, and strategies to address, climate change communications. The course covered strategic principles, including goal setting, designing messages, and selecting channels and sources appropriate for reaching diverse audiences.

To meet the needs of working professionals, the program was designed with both synchronous and asynchronous components to balance flexibility and engagement. Each week, participants asynchronously viewed a 60–90 min video-recorded lecture, broken into 10–25 min segments. Synchronous, live weekly discussion sessions, conducted via Zoom and led by teaching assistant discussion leaders, were staggered throughout the week to accommodate different work schedules and time zones; they brought together relatively small (≤ 15) groups of participants to review the week’s material, connect it to their own context, and encourage peer-to-peer learning. Each week also included readings, a quiz or short assignment, and an optional discussion board. In addition, each course had a concluding assignment. The expectation was that participants would devote an average of 5–7 h per week to the program.

Here we present a mixed-methods evaluation of the first seven cohorts of the program conducted between 2018 and 2022, based on surveys completed by participants after each individual course and after completing the overall certificate program. We utilized both structured (selecting from predetermined responses) and unstructured (free text) response data to review participants’ feedback on the quality of course instruction and material and the relevance of what they learned to their professional and personal lives.

We evaluated this course as a case study to shed light on how educators can provide working professionals with the information and tools they need to effectively address the public health challenges of climate change in their work. Our goal was to identify program strengths and opportunities for program improvement and to share what we learned to enhance and support ongoing efforts of educators to deliver thought- and behavior-changing information on climate change and health to those who are in a position to make a difference.

## Materials and methods

### Ethics approval and consent to participate

The need for ethics approval was waived by the Yale University Institutional Review Board (IRB). The Yale University IRB waived the need for informed consent due to the use of extant data in a retrospective analysis.

### Data collection

Between September 2018 and February 2022, the program was offered seven times to seven different cohorts. During each offering, participants were asked to complete a course evaluation survey after each course (Courses 1–3) and an overall program evaluation after they completed the program, resulting in a total of 28 administered surveys. The surveys were administered via Canvas (a learning management system). Surveys included structured multiple-choice questions and open ended free-text questions. We used Likert-scales (e.g., excellent, very good, good, below average, and poor) for structured questions rating the courses, course instructors, lectures, discussion leaders, discussion sessions, readings, discussion boards, and overall program. Another multiple-choice question queried about the number of hours per week spent on the course and a yes/no question in the overall program evaluation asked whether the respondent would recommend the program. Open-ended questions asked participants to provide feedback on course and program elements (see Supplemental Materials for the course and overall program surveys). We retrieved information on participant characteristics from their applications to the program. This information included primary country of residence, primary state or province of residence, organization of employment, and highest level of education.

### Quantitative evaluation

We used R version 4.2.1 for data cleaning and analysis, which included summary/descriptive statistics and visualizations. Likert-scale response values were changed from categorical “poor” to “excellent” or “strongly disagree” to “strongly agree” to a numeric 1–5 scale and from “not at all important” to “very important” to a numeric 1–4 scale. Survey response rates are presented in Table S1. We assessed predictors of program completion using univariable logistic regression models with 1 indicating completion and 0 indicating non-completion.

### Qualitative evaluation

Spreadsheets with the open-ended responses were uploaded to NVivo for coding. We developed the codebook using an iterative, dynamic process including a preliminary review of the data for notable themes, developing a first draft of the codebook, and then having two researchers code ~ 10% of the data (three course surveys). The researchers reflected on the coding process, added codes corresponding to newly identified themes, and removed those that were ineffective. The codebook was finalized, and the surveys were split evenly between two researchers for coding, which was done using each individual free-text answer response as the unit of analysis. A full list of codes is presented in Table S2.

To evaluate intercoder reliability, we randomly selected a subset of six surveys (~ 20%; four course surveys and two overall program surveys) to be coded by both researchers, and used the NVivo Coding Comparison Query to provide a Cohen’s Kappa statistic for each of the individual codes used across the subset. The mean Kappa score was 0.80, which we deemed sufficient interrater reliability [8]. Codes that fell below a Kappa score of 0.6 can be found in Table S3.

After coding was completed, themes and corresponding exemplary quotes were identified. The analysis was iterative, involving a review of the raw responses, codes, and NVivo visualizations of “nodes” (i.e., common relationships among codes), and identification of recurring points made by respondents. The themes were categorized into two domains: program strengths and opportunities for improvement. Themes were revised as responses were reviewed and were shaped by the content, frequency, applicability, and significance of responses supporting or contradicting each theme. We selected one exemplary quote per theme, with a preference for responses offering actionable feedback.

## Results

### Participant characteristics

Participants of the certificate program resided across six continents and all country income levels, but overwhelmingly resided in North America (82.4%) and high-income countries (90.3%) (Table 1). Most participants held a bachelor’s degree (27.9%) or higher (66.6%). They were relatively evenly distributed according to organization of employment: academic institutions (25.1%), non-profit organizations (19.9%), government (16.6%), and healthcare (16.4%). Cohort-specific participant characteristics are presented in Table S4.

**Table 1:** Characteristics of enrolled students, cohorts 1–7

Characteristic	Frequency (*N* = 579)
**Highest Level of Education** ^**a**^	
Doctoral Degree	96 (18.3%) ^b^
Professional Degree	46 (8.8%)
Master’s Degree	207 (39.5%)
Bachelor’s Degree	146 (27.9%)
2 Years of College or Less	29 (5.5%)
**Region of Primary Residence**	
North America	477 (82.4%)
South/Central America	19 (3.3%)
Asia	22 (3.8%)
Africa	19 (3.3%)
Europe	29 (5.0%)
Oceania	13 (2.2%)
**Country Income Level**	
High-Income	523 (90.3%)
Upper-Middle-Income	24 (4.1%)
Lower-Middle-Income	26 (4.5%)
Low-Income	6 (1.0%)
**Organization of Employment** ^**c**^	
Academic	145 (25.1%)
Business	81 (14.0%)
Government	96 (16.6%)
Healthcare	95 (16.4%)
Non-profit/Non-governmental Organization	115 (19.9%)
Other	46 (8.0%)
**Program Completion Rate**	
Completed Certificate	521 (90.0%)

^a^ 55 non-responses. Participants were asked to write in the field of their highest degree in a separate response. We observed inconsistencies in responses such that some participants with certain doctoral degrees (e.g., MD, JD) identified their degree as a Professional Degree, while others in the same field identified their degree as a Doctoral Degree.

^b^ Percentages may not sum to 100.0% due to rounding

^c^ 1 non-response

### Quantitative evaluation

Participants rated the three courses and overall program highly. On a scale from “poor” (coded as 1) to “excellent,” (coded as 5), mean (standard deviation) ratings were 4.42 (0.65), 4.26 (0.81), and 4.41 (0.76) for Courses 1–3, respectively (Tables S5-S7). The overall program was rated higher, with a mean rating of 4.62 (0.59) and 98.6% of participants reporting they would recommend the program to others (Table 2). Respondents also gave high ratings to each course director (4.45 [0.69], 4.41 [0.74], and 4.48 [71], respectively) and to each discussion leader (4.52 [0.70], 4.70 [0.56], and 4.42 [0.81], respectively) (Tables S5-S7).

**Table 2:** Overall program ratings (mean [standard deviation], unless otherwise noted), by cohort

Cohort:	1	2	3	4	5	6	7	Overall
	(*N* = 53)	(*N* = 68)	(*N* = 62)	(*N* = 76)	(*N* = 76)	(*N* = 75)	(*N* = 78)	(*N* = 488)
Overall program rating (maximum score is 5) ^a^	4.58 (0.63)	4.75 (0.47)	4.60 (0.56)	4.67 (0.53)	4.55 (0.68)	4.63 (0.59)	4.56 (0.62)	4.62 (0.59)
Would recommend the program to others (% responding yes)	100%	100%	98.4%	98.7%	98.7%	97.3%	97.4%	98.6%
**The maximum score for the following items is 4**: ^**b**^								
Importance of lectures	-	3.82 (0.42)	3.81 (0.51)	3.74 (0.50)	3.75 (0.49)	3.70 (0.57)	3.74 (0.50)	3.76 (0.50)
Importance of assignments	-	3.43 (0.72)	3.42 (0.74)	3.43 (0.79)	3.24 (0.85)	3.34 (0.82)	3.65 (0.58)	3.42 (0.76)
Importance of readings	-	2.85 (0.76)	2.90 (0.78)	2.72 (0.74)	2.96 (0.76)	2.86 (0.78)	2.72 (0.75)	2.83 (0.76)
Importance of live discussions	-	3.24 (0.87)	3.11 (0.85)	3.34 (0.81)	3.28 (0.84)	3.43 (0.76)	3.53 (0.72)	3.33 (0.81)
Importance of optional discussion boards	-	2.28 (0.88)	1.63 (0.81)	1.95 (0.73)	1.78 (0.81)	1.82 (0.80)	1.99 (0.80)	1.91 (0.82)

^a^ Values are from Likert-scale response values, where 1 = “poor,” 2 = “below average,” 3 = “good,” 4 = “very good,” and 5 = “excellent.”

^b^ Values are from Likert-scale response values, where 1 = “not at all important,” 2 = “somewhat important,” 3 = “important,” and 4 = “very important.” Cohort 1 was not asked these questions.

When asked on a four-level scale from “not at all important” (coded as 1) to “very important” (coded as 4) about the importance of each learning activity to their mastery of the course material, respondents gave a mean rating of 3.76 (0.50) for the lectures, followed by the assignments (3.42 [0.76]), live discussions (3.33 [0.81]), assigned readings (2.83 [ 0.76]), and discussion boards (1.91 [0.82]), which was the only optional activity (Table 2). The modal amount of time spent per week on each course was 5–7 h (Figures S1-S3).

The certificate program was completed by 90.0% of enrollees (Table 1). We explored the relationship between enrollee characteristics and certificate program completion (Table S8). Participants working in businesses had a higher odds of completion compared to those from academic institutions (OR = 4.40, 95% CI = 1.46–19.1), and participants in Cohort 4 had a higher odds of completion compared to those in Cohort 1 (OR = 3.41, 95% CI = 1.17–11.3). We saw no differences by education level, region of primary residence, or country income level.

### Qualitative evaluation

We identified themes and separated them into two domains: (1) the strengths of the program and (2) opportunities for improvement. The codes related to each theme can be found in Table S9.

#### Themes related to program strengths

**Theme 1: Valuable curriculum.** Participants reported the importance, applicability, and value of the program’s content.

**Exemplary quote**: *“I valued the program’s focus on practical application of health behavior and health communications strategies to the issue of climate change. I also appreciated the program’s focus on thinking through solutions that could be applied in the participant’s local context. I found it meaningful to reflect on how climate change is already impacting and will continue to impact people in all areas of the world*,* and to be prompted to think about climate change risk as a social determinant of health. I felt that the three courses were offered in the right order to maximize understanding and to prompt action.”*

**Theme 2: Peer-to-peer learning.** Participants reported an appreciation for networking and horizontal learning opportunities.

**Exemplary quote**: *“I greatly enjoyed the discussions since our participants came from a variety of backgrounds and could offer perspectives on readings*,* lectures*,* and ideas that was indispensable to my understanding of the course material. I’ve made many contacts and friendships in the program that have influenced my understandings and perspectives.”*

**Theme 3: A call to action.** Participants reported galvanization towards action at the intersection of climate change and human health.

**Exemplary quote**: *“This course was very encouraging and speaks to the reality that we all need to do our part to get the message out*,* to repeat that message*,* to understand our audience and to move forward to heal the earth. We cannot wait for a top-down*,* federal mandate*,* although we are getting closer. The difference will be made locally*,* and personally with many grassroots movements which require many trained climate change leaders. This course provides the necessary information and skills for grassroots climate change leaders.”*

Numerous respondents reflected on the importance and applicability of content throughout the 18 weeks of the program (Theme 1: Valuable curriculum). They appreciated the sequence of the curriculum, with a broad overview of the field in the first course (Introduction to Climate Change and Health), and then the following courses that prompted real-world application: Climate Adaptation for Human Health and Communicating Climate Change and Health. Participants were largely working professionals from diverse professions, geographies, and life experiences, and they highlighted the importance of learning not only from the curriculum, but from their peers (Theme 2: Peer-to-peer learning). This horizontal learning was facilitated by live discussions guided by a discussion leader. Discussion leaders promoted active engagement with the course material to clarify the scientific content, methodologies, and policy implications, but also provided ample opportunity to ground these lessons with lived experience. For many participants, course content and peer learning led to a sense of responsibility towards action in their sphere of influence (Theme 3: A call to action). Diverse levels of baseline knowledge meant that many participants were unfamiliar with the pace of climate change, the scope of its (potential) impacts, or the co-benefits of action. Orientation to these concepts prompted participants to consider how to make change in their personal or professional lives, on which they often focused in their course assignments.

#### Themes related to opportunities for improvement

**Theme 4: Irrelevant course content.** Participants reported that lessons may be too basic or not applicable to themselves or their context.

**Exemplary quote (specifically about Course 2, Climate Adaptation for Human Health)**: *“I understood the build of the course over one concept to get to a final adaptation project*,* but it all felt really basic and did not provide learning opportunities outside of basic project mgt… I would have liked more focus on collective problem-solving of current issues or learning more about other applied climate change adaptations than so much focus on the one adaptation exercise*,* which left me wanting. Lecture time used to cover project mgt tools did not feel like a good use of time. I don’t feel this course is setting me up with skills that would help pivot my career to something environmentally-focused.”*

**Theme 5: Challenges with course structure.** Participants reported challenges with the course structure, format, and/or volume of content.

**Exemplary quote (specifically about Course 3**,** Communicating Climate Change and Health)**: *“I do think that there was too much material crammed into the short 6-week course. Aside from adapting the content so that it better fits into the 6-week time frame*,* or expanding the course to 8–10 weeks*,* there’s one other change that I can recommend though I’m not sure how this might be done. I thought (on more than one occasion) that it would have been useful (perhaps more useful) to have the course content from the second course and this third course more blended*,* rather than be presented consecutively. That might have helped with the comprehension of some of the material.”*

**Theme 6: Insufficient opportunities for networking.** Participants reported the need for more room for peer collaboration and networking.

**Exemplary quote**: *“I was hoping for more networking opportunities– I found it was very hard to connect during the live sessions… I think there were missed opportunities that utilizing breakout rooms*,* check-in questions*,* and other tips could have helped (even asking participants to rename with their location)*,* utilizing the chat function… all in an effort to promote more peer to peer discussion and networking.”*

Responses under this domain were focused on opportunities for improvement, including references to course content that was either not relevant or not useful to a particular participant’s goals or geographic region (Theme 4: Irrelevant course content). These responses, which were complementary to Theme 1 (Valuable curriculum), reflected the overall challenge of designing a program for diverse professionals with heterogeneous backgrounds and training, as well as disparate geopolitical contexts globally. Theme 5 (Challenges with course structure) highlighted this challenge from a different vantage point including potentially dense learning materials and objectives. The program made few assumptions on background knowledge and therefore packed substantial material into relatively dense six-week courses. Some participants suggested modifying the length or formatting of the three courses to improve learning. Finally, complementary to Theme 2 (Peer-to-peer learning), were mentions of wanting more peer-to-peer networking in Theme 6 (Insufficient opportunities for networking). However, rather than just being focused on peer learning, this theme highlighted the desire for networking from a professional perspective. Many responses indicated a career shift, meaning the respondent discussed plans or hopes to apply the certificate material to shifting their career or job focus. The participants also spanned early-career to senior-level professionals. Therefore, it is easy to understand why participants would desire networking opportunities within their cohort.

## Discussion

This paper presents an assessment of the Yale Climate Change and Health Certificate program for working professionals, leveraging course and program evaluation surveys across seven cohorts. To the best of our knowledge, this program was one of the first of its kind -- an online non-degree program in climate change and health. Our findings suggest a substantial niche to educate a growing number of professionals at the nexus of climate change and health, including individuals who are pivoting their careers into this area [5]. This study identified many strengths and opportunities for improvement using both quantitative and qualitative approaches.

Our quantitative evaluation indicated a high level of satisfaction with the overall program, each of the three courses, the instructors, and the discussion leaders, with 98.6% of participants reporting they would recommend the program to others. Among the program strengths identified in the qualitative evaluation were strong course content and instruction, thoughtful assignments that supported learning, skilled facilitation of discussion groups, and a related emphasis on peer-to-peer learning. Discussion sections particularly reinforced learning by providing personal narratives and/or experiential entry points for other participants [9]. Participants recognized that climate change is potentially the most pressing global public health issue of our time; we need more dedicated professionals across civil society to be involved in mitigating and adapting to the health impacts of climate change. Weaknesses of the program included some mixed reviews on the usefulness of course material (both based on past knowledge and global representation), limited utility of online discussion boards, and insufficient opportunities for more professional networking within the cohorts. Increasing the representation of participants from low- and middle-income countries (LMICs) represents an additional opportunity for improvement, along with increasing the focus of examples and case studies to areas outside of North America and Europe.

Soon after these cohorts, the program was revamped under new direction. Utilizing the data from these evaluations, we made several key improvements and adaptations to the program’s administration and curriculum. We have made a concerted effort to prioritize representation of participants from LMICs by creating tiered tuition categories based on the country of residence. Further, we have worked to improve the applicability of the curriculum by using more examples throughout the global south and LMICs, broadening our use of language and conceptual models for wider-applicability, and encouraging participants to bring news articles or case studies to discussion sections for group discussion. Starting with Cohort 10 of the program, we have included region-specific “short courses” nested between Course 1 and Course 2. Each participant enrolls in one of these three-week (as opposed to six-week ) courses, which are taught by regional experts and focus on climate impacts and/or mitigation opportunities in South Asia, Sub-Saharan Africa, and the Caribbean, respectively. These regions were chosen based on established institutional relationships and disproportionate climate impacts and social vulnerability [10, 11, 12].

This study had numerous strengths including evaluations from seven cohorts, a mixed-methods approach that allowed us to explore themes not captured in structured data, and a high response rate. There are few other primary educational evaluations of educational offerings on climate change and health [3, 13, 14], a topic of increasing importance for public health. While this was a robust evaluation, there were notable limitations. Many participants provided terse or abbreviated responses to the free-text questions and results may be biased due to differences between respondents and nonrespondents. Finally, we have not conducted follow up surveys of program alumni so are unable to assess long-term trajectories after the program. Education is meant to induce behavior change through the provision of knowledge and skills, but the degree to which behavior change takes place can only be evaluated longitudinally. Future studies would benefit from longitudinal tracking of certificate alumni, so we are currently developing a follow up survey to contact the participants and learn more about their (1) professional development; (2) public engagement; and (3) self-efficacy regarding skills and themes learned through the program.

The impacts of climate change are observed in each region of the planet and involve every element of society in some way; responding to these impacts requires a geographically and professionally diverse workforce to safeguard human health. While climate change and health educational offerings are growing internationally, they are not yet available at the scale required to meet current and future challenges. As we have noted, there is little documentation or consensus on what constitutes appropriate content or pedagogical practice in this field. Nevertheless, our case study shows that diverse working professionals seeking climate change and health education valued strong, relevant course content, professional networking and peer-to-peer learning, and acquisition of effective skills for use in their work. We encourage other climate change and health education programs to conduct similar case studies that, along with ours, will convey lessons learned and catalyze and improve future programs and maximize their utility to society. Ultimately, a workforce (beyond public health) that is more educated on these issues is a necessary pillar to help society weather climate change.

## Conclusions

Our case study demonstrates the value of this non-degree program for participants from diverse professional and academic backgrounds wishing to expand their knowledge at the nexus of climate change and health. As a whole, participants rated the educational offering highly and found utility of the materials in their personal and professional lives. Opportunities exist to continue to develop and hone the curriculum, diversify the geographic representation of the participants, and use digital educational tools more effectively.

## Electronic supplementary material

Below is the link to the electronic supplementary material.

Supplementary Material 1

## Data Availability

Data collected for the study, including individual participant data, will not be shared. The data were from extant educational data; therefore, consent was not provided for sharing. Please contact the corresponding author, daniel.carrion@yale.edu, for more information.
