# Supplementary material for: A mixed methods evaluation of an online climate change and health certificate program for working professionals
Source: BMC Public Health. 2025 Jul 3;25:2308. doi: 10.1186/s12889-025-23477-7 (PMC12224474; doi:10.1186/s12889-025-23477-7)
Supplement: Supplementary file 1 — Supplementary Material 1 [file 12889_2025_23477_MOESM1_ESM.docx]

**Supplemental materials**

**Survey Questions**

***Survey questions for Course evaluations***

Question 1: What is your **overall assessment** of this course?

- Poor
- Below average
- Good
- Very good
- Excellent

Question 2: Looking back on this course, what is your **overall assessment** of the course: What are its strengths and weaknesses, and in what ways might it be improved?

|  |
| --- |

Question 3: What is your overall rating of the **course director**?

- Poor
- Below average
- Good
- Very good
- Excellent

Question 4:

The course director's lectures were clearly and logically presented. [Select from drop down]

- Strongly disagree
- Disagree
- Neutral
- Agree
- Strongly agree

The lectures were presented at an optimal level of difficulty. [Select from drop down]

- Strongly disagree
- Disagree
- Neutral
- Agree
- Strongly agree

The lectures raised questions or problems that encouraged me to think critically. [Select from drop down]

- Strongly disagree
- Disagree
- Neutral
- Agree
- Strongly agree

Question 5: Please **evaluate the course director**. What are the course director's strengths and weaknesses, and in what ways might his or her teaching be improved?

|  |
| --- |

Question 6: What is your overall rating of the **discussion leader**?

- Poor
- Below Average
- Good
- Very good
- Excellent

Question 7:

The discussion leader established a positive learning environment. [Select from drop down]

- Strongly disagree
- Disagree
- Neutral
- Agree
- Strongly agree

The discussion leader raised questions or problems that encouraged me to think critically. [Select from drop down]

- Strongly disagree
- Disagree
- Neutral
- Agree
- Strongly agree

The discussion leader was helpful in facilitating my participation in the course. [Select from drop down]

- Strongly disagree
- Disagree
- Neutral
- Agree
- Strongly agree

Question 8: Please **evaluate the discussion leader**. Please indicate strengths and weaknesses and how his or her facilitation of discussions might be improved.

|  |
| --- |

Question 9: Indicate the total number of **hours per week** you spent on the course.

- 1 to 3
- 3 to 5
- 5 to 7
- 7 to 10
- More than 10

Question 10: What was one major takeaway that you will bring to your work?

|  |
| --- |

Question 11: How might the content have been more relevant to you professionally?

|  |
| --- |

Question 12: How would you **summarize this course** for a fellow student? Would you recommend it to another student? Why or why not?

|  |
| --- |

***Survey questions for full program evaluation***

Question 1: What is your **overall rating** of the Climate Change and Health Certificate?

- Excellent
- Very Good
- Good
- Below Average
- Poor

Question 2: Please tell us what you found **most** useful about the program. As we update the program for the next cohort, what components of the program should we **be sure to retain**?

|  |
| --- |

Question 3: Please tell us what you found **least** useful about the program. As we update the program for the next cohort, are there any aspects we should consider **removing**?

|  |
| --- |

Question 4 *[included only for cohorts 2-7*]: Please indicate how important each of the following learning activities were to your mastery of the course material:

Lectures [select from drop down]

- Not at all important
- Somewhat important
- Important
- Very important

Assignments [select from drop down]

- Not at all important
- Somewhat important
- Important
- Very important

Readings [select from drop down]

- Not at all important
- Somewhat important
- Important
- Very important

Live discussions [select from drop down]

- Not at all important
- Somewhat important
- Important
- Very important

Discussion boards [select from drop down]

- Not at all important
- Somewhat important
- Important
- Very important

Question 5 [*included only for cohorts 2-7*]: Did you experience any barriers to fully participating in the program (e.g., reviewing all lectures and readings, attending weekly live discussions, participating in discussion boards, and completing assignments on time)? If yes, please 1) describe the barriers your experienced and 2) share any suggestions you have for how we might better support future students to overcome these barriers.

|  |
| --- |

Question 6: Would you recommend the program to others?

- Yes
- No

Question 7: If you would recommend the program to other participants, please let us know why. If you would not recommend it, please let us know why not.

|  |
| --- |

Question 8 [*included only for cohort 1*]: An important aspect of the program is the network of peers you have formed with other participants. As the program continues to grow, we would like your input on how best to ensure those who have completed the program remain connected to each other and develop connections with future cohorts.

Please select any of the options below to let us know how you would like to stay connected to others in the program.

- Email listserv
- Participant directory
- Webinars on new developments
- In-person CCH Certificate alumni events
- Newletters
- LinkedIn Group
- Digital CCH Certificate alumni events
- Other

Question 9 [*included only for cohort 1*]: If you have a suggestion for growing the professional network of participants that we have not included above, please share it here:

|  |
| --- |

Question 10 [*included only for cohort 1*]: Please share anything else you would like us to know about your experience in the program.

|  |
| --- |

**Table S1.** Response rates by cohort and course (total responses over total enrolled in cohort^a^)

| Cohort | Course 1 | Course 2 | Course 3 | Full Program |
| --- | --- | --- | --- | --- |
| 1 (N=62) | 85.5% | 79.0% | 83.9% | 85.5% |
| 2 (N=82) | 100% | 90.2% | 78.0% | 82.9% |
| 3 (N=80) | 100% | 91.3% | 60.0% | 77.5% |
| 4 (N=84) | 100% | 100% | 88.1% | 90.5% |
| 5 (N=98) | 94.9% | 92.9% | 85.7% | 77.5% |
| 6 (N=84) | 100% | 96.4% | 88.1% | 89.3% |
| 7 (N=89) | 98.9% | 93.3% | 89.9% | 87.6% |

^a^Total enrolled in cohort includes those who did not complete the program and therefore did not complete one or more surveys.

**Table S2:** Full list of qualitative codes.

| Accessibility |
| --- |
| Applications |
| Audience |
| Balance |
| Career shift |
| Climate change and health connection |
| Collaboration |
| Communications |
| Continued learning |
| Continued network |
| Course content |
| Course structure |
| Difficulty |
| Director feedback |
| Discussion leader feedback |
| Diversity |
| Empowerment |
| Flow |
| Mental shift |
| Mentorship |
| Network |
| New tools |
| Pacing |
| Perspectives |
| Pessimism |
| Program content |
| Program structure |
| Relevance |
| Scope |
| Solutions wanted |
| Supplemental |
| Urgency |
| Value |
| Vulnerable populations |

**Table S3.** Qualitative codes with low (<0.6) Cohen’s Kappa measures and corresponding *post hoc* assessment of discrepancies

| **Code (Kappa)** | **Post hoc review** |
| --- | --- |
| Applications (0.47) | Coders identified a discrepancy in understanding what constituted an application. Research assistant #1 (RA1) coded responses that included mentions of vague applications to work/life while RA2 coded responses that were relatively more concrete in the ways a respondent could apply the course content in their professional roles. |
| Audience (0.59) | There were different approaches to determining what constituted a response about the appropriate course audience. Both RAs included responses that recommended the course to a specific type of person (level of experience, industry, etc.). but only RA1 included responses where a respondent noted that the course wasn’t a good fit for them because of their own professional role or experiences. |
| Communications (0.39) | In retrospect, this code was too broad and there was a discrepancy in how explicit the mention of “communication” should be in order to be coded. RA2 coded responses that indirectly spoke to/described communication strategies while RA1 coded responses that explicitly mentioned communication. |
| Course content (0.46) | In retrospect, this code was too broad. RA1 generally coded responses that gave an opinion/assessment on the course content while RA2 generally coded responses that mentioned the course content in any capacity. |
| Difficulty (0.40) | Both RAs approached ‘difficulty’ by coding level of challenge in the content. However, RA1 took a more binary approach, coding responses that rated the content as challenging or easy while RA2 took a broader approach that included middle-ground responses that spoke to the appropriateness of the content’s challenge level. |
| New tools (0.51) | RAs were consistent in coding responses that explicitly mentioned tools, but there was a discrepancy in coding mentions of “skills” or knowledge learned. RA1 coded responses that vaguely mentioned that skills were generally learned while RA2 coded responses that mentioned a specific skill that was learned. |
| Program structure (0.54) | In retrospect, this code was too broad and there was a discrepancy in what was considered a structural element of the program that should be included. Compared to RA1, RA2 coded more responses that provided feedback on the discussion sections as an element of the program structure. |
| Value (0.49) | This code was used relatively infrequently, so the estimate of Kappa was unstable, with a small discrepancy in coding having a large effect on Kappa. Both RAs approached this code as capturing responses that spoke to financial value; e.g., participants getting their money’s worth. |

**Table S4.** Characteristics of enrolled students by cohort.

|  | **1 (N=62)** | **2 (N=82)** | **3 (N=80)** | **4 (N=84)** | **5 (N=98)** | **6 (N=84)** | **7 (N=89)** | **Overall (N=579)** |
| --- | --- | --- | --- | --- | --- | --- | --- | --- |
| **Highest Level of Education^a^** |  |  |  |  |  |  |  |  |
| Doctoral Degree | 14 (22.6%)^b^ | 17 (20.7%) | 11 (13.8%) | 17 (20.2%) | 19 (19.4%) | 8 (9.5%) | 10 (11.2%) | 96 (16.6%) |
| Professional Degree | 7 (11.3%) | 8 (9.8%) | 9 (11.3%) | 4 (4.8%) | 8 (8.2%) | 4 (4.8%) | 6 (6.7%) | 46 (7.9%) |
| Master's Degree | 30 (48.4%) | 24 (29.3%) | 26 (32.5%) | 47 (56.0%) | 33 (33.7%) | 17 (20.2%) | 30 (33.7%) | 207 (35.8%) |
| Bachelor's Degree | 10 (16.1%) | 25 (30.5%) | 29 (36.3%) | 14 (16.7%) | 30 (30.6%) | 15 (17.9%) | 23 (25.8%) | 146 (25.2%) |
| 2 Years of College or Less | 1 (1.6%) | 5 (6.1%) | 5 (6.3%) | 2 (2.4%) | 4 (4.1%) | 4 (4.8%) | 8 (9.0%) | 29 (5.0%) |
| No Response | 0 (0%) | 3 (3.7%) | 0 (0%) | 0 (0%) | 4 (4.1%) | 36 (42.9%) | 12 (13.5%) | 55 (9.5%) |
| **Region of Primary Residence** |  |  |  |  |  |  |  |  |
| North America | 49 (79.0%) | 68 (82.9%) | 63 (78.8%) | 77 (91.7%) | 83 (84.7%) | 78 (92.9%) | 59 (66.3%) | 477 (82.4%) |
| South/Central America | 1 (1.6%) | 2 (2.4%) | 4 (5.0%) | 0 (0%) | 1 (1.0%) | 0 (0%) | 11 (12.4%) | 19 (3.3%) |
| Asia | 3 (4.8%) | 2 (2.4%) | 4 (5.0%) | 0 (0%) | 4 (4.1%) | 1 (1.2%) | 8 (9.0%) | 22 (3.8%) |
| Africa | 5 (8.1%) | 7 (8.5%) | 2 (2.5%) | 3 (3.6%) | 0 (0%) | 0 (0%) | 2 (2.2%) | 19 (3.3%) |
| Europe | 2 (3.2%) | 2 (2.4%) | 5 (6.3%) | 3 (3.6%) | 6 (6.1%) | 4 (4.8%) | 7 (7.9%) | 29 (5.0%) |
| Oceania | 2 (3.2%) | 1 (1.2%) | 2 (2.5%) | 1 (1.2%) | 4 (4.1%) | 1 (1.2%) | 2 (2.2%) | 13 (2.2%) |
| **Country Income Level** |  |  |  |  |  |  |  |  |
| High-Income | 53 (85.5%) | 74 (90.2%) | 72 (90.0%) | 81 (96.4%) | 94 (95.9%) | 82 (97.6%) | 67 (75.3%) | 523 (90.3%) |
| Upper-Middle-Income | 2 (3.2%) | 1 (1.2%) | 4 (5.0%) | 0 (0%) | 1 (1.0%) | 1 (1.2%) | 15 (16.9%) | 24 (4.1%) |
| Lower-Middle-Income | 5 (8.1%) | 5 (6.1%) | 3 (3.8%) | 3 (3.6%) | 3 (3.1%) | 1 (1.2%) | 6 (6.7%) | 26 (4.5%) |
| Low-Income | 2 (3.2%) | 2 (2.4%) | 1 (1.3%) | 0 (0%) | 0 (0%) | 0 (0%) | 1 (1.1%) | 6 (1.0%) |
| **Organization of Employment** |  |  |  |  |  |  |  |  |
| Academic | 21 (33.9%) | 28 (34.1%) | 22 (27.5%) | 18 (21.4%) | 23 (23.5%) | 15 (17.9%) | 18 (20.2%) | 145 (25.0%) |
| Business | 6 (9.7%) | 5 (6.1%) | 10 (12.5%) | 14 (16.7%) | 19 (19.4%) | 16 (19.0%) | 11 (12.4%) | 81 (14.0%) |
| Government | 15 (24.2%) | 5 (6.1%) | 21 (26.3%) | 20 (23.8%) | 13 (13.3%) | 10 (11.9%) | 12 (13.5%) | 96 (16.6%) |
| Healthcare | 4 (6.5%) | 19 (23.2%) | 11 (13.8%) | 16 (19.0%) | 21 (21.4%) | 17 (20.2%) | 7 (7.9%) | 95 (16.4%) |
| Non-profit/Non-governmental organization | 13 (21.0%) | 18 (22.0%) | 11 (13.8%) | 10 (11.9%) | 12 (12.2%) | 18 (21.4%) | 33 (37.1%) | 115 (19.9%) |
| Other | 3 (4.8%) | 7 (8.5%) | 5 (6.3%) | 6 (7.1%) | 10 (10.2%) | 8 (9.5%) | 7 (7.9%) | 46 (7.9%) |
| No Response | 0 (0%) | 0 (0%) | 0 (0%) | 0 (0%) | 0 (0%) | 0 (0%) | 1 (1.1%) | 1 (0.2%) |
| **Program Completion Rate** |  |  |  |  |  |  |  |  |
| Completed Certificate | 51 (82.3%) | 70 (85.4%) | 70 (87.5%) | 79 (94.0%) | 87 (88.8%) | 75 (89.3%) | 89 (100%) | 521 (90.0%) |

^a^ Participants were asked to write in the field of their highest degree in a separate response. We observed inconsistencies in responses such that some participants with certain doctoral degrees (e.g., MD, JD) identified their degree as a Professional Degree, while others in the same field identified their degree as a Doctoral Degree. ^b^ Percentages may not sum to 100.0% due to rounding.

**Table S5.** Course 1 ratings (mean [standard deviation]), by cohort. Maximum score is 5.

|  | **Cohort** | | | | | | |  |
| --- | --- | --- | --- | --- | --- | --- | --- | --- |
|  | **1** | **2** | **3** | **4** | **5** | **6** | **7** | **Overall** |
|  | **(N=53)** | **(N=82)** | **(N=80)** | **(N=84)** | **(N=93)** | **(N=87)** | **(N=88)** | **(N=567)** |
| Overall assessment of course^a^ | 4.34 (0.68) | 4.46 (0.65) | 4.28 (0.69) | 4.39 (0.62) | 4.47 (0.67) | 4.48 (0.63) | 4.48 (0.62) | 4.42 (0.65) |
| Overall rating of course director^a^ | 4.42 (0.75) | 4.45 (0.69) | 4.24 (0.73) | 4.42 (0.73) | 4.51 (0.67) | 4.55 (0.61) | 4.55 (0.64) | 4.45 (0.69) |
| Lectures clearly and logically presented^b^ | 4.60 (0.69) | 4.62 (0.66) | 4.55 (0.50) | 4.58 (0.50) | 4.70 (0.59) | 4.72 (0.60) | 4.56 (0.83) | 4.62 (0.63) |
| Lectures presented at an optimal level of difficulty^b^ | 4.21 (0.74) | 4.27 (0.83) | 4.28 (0.59) | 4.37 (0.6) | 4.38 (0.81) | 4.42 (0.73) | 4.07 (1.13) | 4.29 (0.80) |
| Lectures encouraged critical thinking^b^ | 4.45 (0.85) | 4.46 (0.76) | 4.41 (0.63) | 4.46 (0.55) | 4.44 (0.77) | 4.66 (0.57) | 4.53 (0.79) | 4.49 (0.70) |
| Overall rating of discussion leader^a^ | 4.43 (0.72) | 4.51 (0.63) | 4.53 (0.69) | 4.63 (0.65) | 4.57 (0.63) | 4.40 (0.86) | 4.52 (0.68) | 4.52 (0.70) |
| Discussion leader established positive learning environment^b^ | 4.53 (0.87) | 4.67 (0.67) | 4.78 (0.44) | 4.76 (0.69) | 4.73 (0.77) | 4.78 (0.52) | 4.69 (0.63) | 4.72 (0.66) |
| Discussion leader encouraged critical thinking^b^ | 4.36 (0.96) | 4.60 (0.54) | 4.30 (0.72) | 4.40 (0.75) | 4.22 (0.85) | 4.29 (0.96) | 4.40 (0.70) | 4.36 (0.79) |
| Discussion leader facilitated participation^b^ | 4.57 (0.57) | 4.51 (0.72) | 4.62 (0.65) | 4.56 (0.80) | 4.55 (0.80) | 4.59 (0.76) | 4.60 (0.63) | 4.57 (0.72) |

^a^ Values are from Likert-scale response values, where 1 = “poor,” 2 = “below average,” 3 = “good,” 4 = “very good,” and 5 = “excellent.”
^b^ Values are from Likert-scale response values, where 1 = “strongly disagree,” 2 = “disagree,” 3 = “neutral,” 4 = “agree,” and 5 = “strongly agree.”

**Table S6.** Course 2 ratings (mean [standard deviation]), by cohort. Maximum score is 5.

|  | **Cohort** | | | | | | |  |
| --- | --- | --- | --- | --- | --- | --- | --- | --- |
|  | **1** | **2** | **3** | **4** | **5** | **6** | **7** | **Overall** |
|  | **(N=49)** | **(N=74)** | **(N=73)** | **(N=84)** | **(N=91)** | **(N=81)** | **(N=83)** | **(N=535)** |
| Overall assessment of course^a^ | 4.37 (0.78) | 4.46 (0.67) | 3.96 (0.93) | 4.32 (0.7) | 4.21 (0.77) | 4.22 (0.87) | 4.31 (0.85) | 4.26 (0.81) |
| Overall rating of course director^a^ | 4.46 (0.65) | 4.53 (0.80) | 4.22 (0.82) | 4.45 (0.65) | 4.35 (0.77) | 4.43 (0.71) | 4.42 (0.75) | 4.41 (0.74) |
| Lectures clearly and logically presented^b^ | 4.65 (0.56) | 4.64 (0.51) | 4.36 (0.73) | 4.54 (0.61) | 4.31 (0.90) | 4.44 (0.63) | 4.47 (0.75) | 4.47 (0.70) |
| Lectures presented at an optimal level of difficulty^b^ | 4.12 (1.16) | 4.35 (0.81) | 4.05 (0.88) | 4.26 (0.79) | 4.13 (0.92) | 4.17 (0.75) | 4.04 (1.06) | 4.16 (0.91) |
| Lectures encouraged critical thinking^b^ | 4.43 (0.74) | 4.48 (0.71) | 4.07 (0.89) | 4.42 (0.70) | 4.21 (0.77) | 4.38 (0.74) | 4.34 (0.90) | 4.32 (0.79) |
| Overall rating of discussion leader^a^ | 4.60 (0.68) | 4.73 (0.58) | 4.34 (0.75) | 4.75 (0.49) | 4.81 (0.47) | 4.77 (0.43) | 4.82 (0.45) | 4.70 (0.56) |
| Discussion leader established positive learning environment^b^ | 4.77 (0.47) | 4.77 (0.68) | 4.67 (0.50) | 4.85 (0.36) | 4.79 (0.55) | 4.89 (0.32) | 4.86 (0.39) | 4.80 (0.48) |
| Discussion leader encouraged critical thinking^b^ | 4.46 (0.74) | 4.56 (0.75) | 4.21 (0.80) | 4.68 (0.49) | 4.61 (0.67) | 4.75 (0.43) | 4.77 (0.50) | 4.59 (0.65) |
| Discussion leader facilitated participation^b^ | 4.65 (0.60) | 4.74 (0.67) | 4.52 (0.67) | 4.77 (0.45) | 4.72 (0.65) | 4.8 (0.56) | 4.86 (0.39) | 4.73 (0.58) |

^a^ Values are from Likert-scale response values, where 1 = “poor,” 2 = “below average,” 3 = “good,” 4 = “very good,” and 5 = “excellent.”
^b^ Values are from Likert-scale response values, where 1 = “strongly disagree,” 2 = “disagree,” 3 = “neutral,” 4 = “agree,” and 5 = “strongly agree.”

**Table S7.** Course 3 ratings (mean [standard deviation]), by cohort. Maximum score is 5.

|  | **Cohort** | | | | | | |  |
| --- | --- | --- | --- | --- | --- | --- | --- | --- |
|  | **1** | **2** | **3** | **4** | **5** | **6** | **7** | **Overall** |
|  | **(N=52)** | **(N=64)** | **(N=48)** | **(N=74)** | **(N=84)** | **(N=74)** | **(N=80)** | **(N=476)** |
| Overall assessment of course^a^ | 4.42 (0.85) | 4.55 (0.64) | 4.48 (0.74) | 4.61 (0.66) | 4.38 (0.77) | 4.28 (0.80) | 4.24 (0.82) | 4.41 (0.76) |
| Overall rating of course director^a^ | 4.50 (0.73) | 4.62 (0.66) | 4.48 (0.62) | 4.59 (0.66) | 4.44 (0.72) | 4.42 (0.81) | 4.35 (0.71) | 4.48 (0.71) |
| Lectures clearly and logically presented^b^ | 4.67 (0.55) | 4.62 (0.66) | 4.60 (0.49) | 4.68 (0.50) | 4.56 (0.77) | 4.49 (0.80) | 4.55 (0.53) | 4.59 (0.63) |
| Lectures presented at an optimal level of difficulty^b^ | 4.25 (0.99) | 4.46 (0.86) | 4.44 (0.65) | 4.49 (0.71) | 4.38 (0.81) | 4.26 (0.98) | 4.20 (0.83) | 4.35 (0.84) |
| Lectures encouraged critical thinking^b^ | 4.56 (0.57) | 4.56 (0.74) | 4.52 (0.55) | 4.65 (0.61) | 4.49 (0.80) | 4.38 (0.93) | 4.49 (0.62) | 4.52 (0.71) |
| Overall rating of discussion leader^a^ | 4.48 (0.78) | 4.66 (0.51) | 4.56 (0.77) | 4.74 (0.55) | 4.26 (0.81) | 4.04 (0.97) | 4.30 (0.89) | 4.42 (0.81) |
| Discussion leader established positive learning environment^b^ | 4.65 (0.68) | 4.83 (0.42) | 4.81 (0.53) | 4.92 (0.27) | 4.51 (0.74) | 4.32 (0.86) | 4.69 (0.61) | 4.66 (0.65) |
| Discussion leader encouraged critical thinking^b^ | 4.54 (0.67) | 4.56 (0.59) | 4.48 (0.68) | 4.69 (0.57) | 4.37 (0.79) | 4.16 (0.95) | 4.35 (0.87) | 4.44 (0.77) |
| Discussion leader facilitated participation^b^ | 4.60 (0.69) | 4.62 (0.55) | 4.73 (0.54) | 4.75 (0.55) | 4.38 (0.81) | 4.19 (0.95) | 4.47 (0.84) | 4.51 (0.76) |

^a^ Values are from Likert-scale response values, where 1 = “poor,” 2 = “below average,” 3 = “good,” 4 = “very good,” and 5 = “excellent.”
^b^ Values are from Likert-scale response values, where 1 = “strongly disagree,” 2 = “disagree,” 3 = “neutral,” 4 = “agree,” and 5 = “strongly agree.”

**Figure S1.** Total hours spent weekly, Course 1


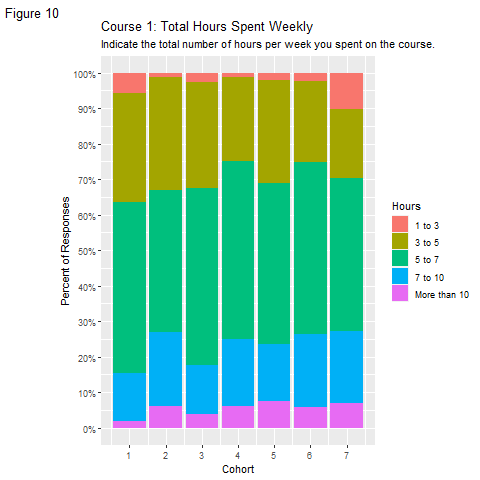


**Figure S2.** Total hours spent weekly, Course 2


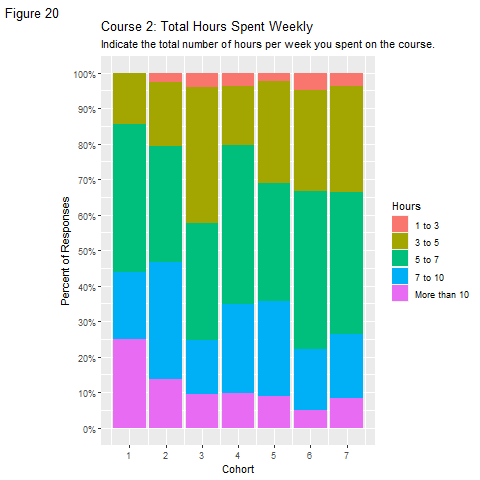


**Figure S3.** Total hours spent weekly, Course 3


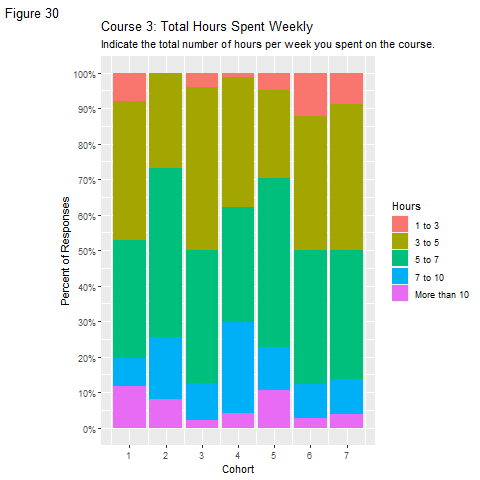


**Table S8.** Univariate associations between enrollee characteristics and completion of the certificate program.

| Characteristic | Odds Ratio | 95% CI | p-value |
| --- | --- | --- | --- |
| Highest Level of Education ^a^ |  |  |  |
| Doctoral Degree | — | — |  |
| Professional Degree | 0.49 | 0.17, 1.40 | 0.2 |
| Master’s Degree | 1.32 | 0.54, 3.09 | 0.5 |
| Bachelor’s Degree | 0.90 | 0.36, 2.12 | 0.8 |
| 2 Years of College or Less | 0.50 | 0.16, 1.74 | 0.2 |
| Region of Primary Residence |  |  |  |
| North America | — | — |  |
| South/Central America ^b^ | NA | NA | NA |
| Asia | 0.68 | 0.22, 2.95 | 0.5 |
| Africa | 0.91 | 0.25, 5.84 | 0.9 |
| Europe | 0.51 | 0.20, 1.58 | 0.2 |
| Oceania | 0.59 | 0.15, 3.87 | 0.5 |
| Country Income Level |  |  |  |
| High-Income | — | — |  |
| Upper-Middle-Income | 2.59 | 0.53, 46.9 | 0.4 |
| Lower-Middle-Income | 0.86 | 0.29, 3.73 | 0.8 |
| Low-Income | 0.56 | 0.09, 10.9 | 0.6 |
| Organization of Employment |  |  |  |
| Academic | — | — |  |
| Business | 4.40 | 1.46, 19.1 | 0.019 |
| Government | 1.19 | 0.56, 2.61 | 0.7 |
| Healthcare | 1.84 | 0.81, 4.60 | 0.2 |
| Non-profit/Non-governmental Organization | 1.60 | 0.75, 3.59 | 0.2 |
| Other | 2.43 | 0.79, 10.6 | 0.2 |
| Cohort |  |  |  |
| 1 | — | — |  |
| 2 | 1.26 | 0.51, 3.09 | 0.6 |
| 3 | 1.51 | 0.59, 3.89 | 0.4 |
| 4 | 3.41 | 1.17, 11.3 | 0.031 |
| 5 | 1.71 | 0.68, 4.26 | 0.2 |
| 6 | 1.80 | 0.70, 4.76 | 0.2 |
| 7 ^b^ | NA | NA | NA |

^a^ Participants were asked to write in the field of their highest degree in a separate response. We observed inconsistencies in responses such that some participants with certain doctoral degrees (e.g. MD, JD) identified their degree as a Professional Degree, while others in the same field identified their degree as a Doctoral Degree.

^b^ Models are not interpretable due to perfect separation, i.e., participants in cohort 7 and participants whose primary country of residence was in South/Central America had 100% completion rates.

**Table S9:** Codes related to and informing the core qualitative themes.

| Theme | Related codes |
| --- | --- |
| 1 | applications, audience, career shift, course content, difficulty, director feedback, empowerment, mental shift, new tools, program content, relevance, value |
| 2 | collaboration, diversity, mentorship, network, perspectives |
| 3 | career shift, empowerment, mental shift, urgency |
| 4 | climate change and health connection, course content, program content, scope, solutions wanted, value |
| 5 | accessibility, balance, course structure, difficulty, director feedback, discussion leader feedback, flow, pacing, program structure, supplemental |
| 6 | continued network, network, perspectives |
